# Pazopanib treatment for primary cardiovascular sarcoma: A retrospective observational study and literature review

**DOI:** 10.3892/ol.2026.15829

**Published:** 2026-08-21

**Authors:** Shintaro Yamanaka, Yoshihiro Yakushijin, Shinji Hasebe, Shoichiro Yamamoto, Tomomi Fujii, Riko Kitazawa, Taketsugu Fujibuchi, Teruki Kidani, Masaki Maruta

**Affiliations:** 1 Cancer Center, Ehime University Hospital, Toon, Ehime 791-0295, Japan; 2 Department of Clinical Oncology, Ehime University Graduate School of Medicine, Toon, Ehime 791-0295, Japan; 3 Palliative Care Center, Ehime University Hospital, Toon, Ehime 791-0295, Japan; 4 Department of Diagnostic Pathology, Ehime University Hospital, Toon, Ehime 791-0295, Japan; 5 Department of Orthopedic Surgery, Ehime University Graduate School of Medicine, Toon, Ehime 791-0295, Japan

**Keywords:** PCS, pazopanib, tyrosine kinase inhibitor, angiosarcoma, intimal sarcoma, leiomyosarcoma

## Abstract

Primary cardiovascular sarcoma (PCS) is a rare, aggressive malignancy with a poor prognosis. The present study describes a single-center experience with pazopanib, an oral multikinase inhibitor, for the outpatient management of unresectable PCS. A retrospective review was conducted of consecutive patients with sarcoma involving the heart and/or pulmonary artery treated with pazopanib at Ehime University Hospital (Toon, Japan) between January 2015 and December 2025. Pazopanib was initiated at 200–400 mg daily and escalated to 600–800 mg according to tolerability. Five patients aged 56–81 years were identified, with angiosarcoma (n=1), intimal sarcoma (n=2) or leiomyosarcoma (n=2). Two patients achieved durable stable disease for >500 days. A case of right atrial angiosarcoma with lung metastasis remained stable for 33 months when treated with 800 mg pazopanib; the patient later died of recurrent cerebral infarction. A case of pulmonary artery intimal sarcoma remained stable for 22 months when treated with 400–600 mg pazopanib; however, treatment was discontinued because of fatigue. Two additional patients stopped treatment because dose escalation led to intolerable toxicities. One patient continued therapy with 800 mg pazopanib as of 8 months, with stable disease, despite requiring a pericardial window for malignant pericardial effusion during treatment. In conclusion, pazopanib can provide meaningful disease control and long-term stabilization for selected patients with unresectable PCS. As treatment-related toxicities frequently limit therapy, individualized dose management and proactive supportive care are essential to maintain treatment continuity and to maximize clinical benefit.

## Introduction

Primary cardiovascular sarcoma (PCS) is an extremely rare malignant tumor that originates in the heart and its associated blood vessels, including the great vessels such as the pulmonary arteries (1). Among 747 reported cases of malignant cardiac tumors, the majority (88.5%) are primary sarcomas. Even in surgically resectable cases, the prognosis is extremely poor, with 30-day and 1-year overall survival (OS) rates of 81.2 and 45.3%, respectively, in this cohort (2). In contrast, there are no detailed reports on the prognosis of unresectable cases treated with chemotherapy, and given the current lack of effective antitumor agents, the prognosis with chemotherapy appears to be similarly poor (2). In the absence of prospective clinical trials specific to PCS, systemic therapy for unresectable disease is generally extrapolated from regimens used for advanced soft tissue sarcoma (STS) (3,4). Because PCS directly involves cardiovascular structures and cardiovascular comorbidities frequently coexist, careful cardiac assessment is warranted when anthracycline-based chemotherapy is considered (3); oral non-anthracycline treatments are therefore of clinical interest for selected patients. PCS may also cause serious cardiovascular complications, including pericardial effusion and embolic events (5,6). Because unresectable PCS is associated with an extremely poor prognosis, management in the outpatient setting is generally preferred. Pazopanib is an orally administered multitargeted tyrosine kinase inhibitor with demonstrated activity in advanced STS. In the randomized phase II EPAZ trial, progression-free survival (PFS) with pazopanib was non-inferior to that with doxorubicin as first-line treatment for metastatic STS in patients aged ≥60 years (7), and in the PALETTE phase III trial, pazopanib prolonged median PFS compared with placebo after previous chemotherapy (8). Although its efficacy specifically for PCS has not been established, its oral administration and demonstrated activity in advanced STS provide a rationale for its use in selected patients with unresectable or recurrent disease. Recently, the authors encountered cases of unresectable PCS treated with pazopanib, a multikinase inhibitor, and disease control was maintained for more than 500 days in two of these patients. In the present report, five patients with unresectable or recurrent PCS treated with pazopanib are described, and the clinical outcomes and the potential role of pazopanib in the management of PCS are discussed.

## Materials and methods

### Patients with cardiovascular sarcoma

The electronic medical record at Ehime University Hospital (Toon, Japan) was queried to identify all patients managed between January 2015 and December 2025 with unresectable sarcomas presenting *de novo* or as recurrences in the heart and/or pulmonary artery who received oral pazopanib. This was a single-center retrospective observational study. Clinicopathological characteristics, disease prognosis, and treatment outcomes were abstracted and summarized. The present study was conducted in accordance with the Declaration of Helsinki and was approved by the Ethics Committee of Ehime University Hospital (approval no. Ehime2603002).

### Immunohistochemistry

The specimens were fixed in 10% neutral buffered formalin for 24 h, then processed for paraffin embedding and sectioning; after hematoxylin and eosin (H&E) staining, the specimens were examined under light microscopy. Tissue sections (5-µm thick) were deparaffinized with xylene and rehydrated through a series of graded alcohol. Antigen retrieval was carried out by microwave treatment at 350 W for 10 min in 10 mM citrate buffer (pH 6.0). Endogenous peroxidase activity was blocked with 0.3% H_2_O_2_ in methanol. The primary antibodies were applied, and the slides were incubated for 60 min at room temperature. The avidin-biotin-peroxidase complex method was used for immunohistochemical staining with the DAKO Liquid DAB Substrate Chromogen System (cat. no. K3468; Dako; Agilent Technologies, Inc.) according to the manufacturer's instructions. The primary antibodies used were as follows: Rabbit monoclonal anti-cyclin-dependent kinase 4 (CDK4) antibody (D9G3E, cat. no. 12790, 1:200; Cell Signaling Technology, Inc.), mouse monoclonal anti-murine double minute 2 antibody (IF2, cat. no. 33-7100, 1:50; Invitrogen; Thermo Fisher Scientific, Inc.), rabbit monoclonal anti-vascular endothelial growth factor receptor 2 antibody (55B11, cat. no. 2479, 1:500; Cell Signaling Technology, Inc.), rabbit monoclonal anti-platelet-derived growth factor receptor-β antibody (Y92, cat. no. ab32570, 1:50; Abcam), rabbit monoclonal anti-ERG antibody (EPR3864, cat. no. ab92513, 1:1,000; Abcam), rabbit monoclonal anti-c-Kit (CD117) antibody (EP10, ready-to-use; Roche Diagnostics), mouse monoclonal anti-h-caldesmon antibody (cat. no. GA05461-2, ready-to-use; Dako; Agilent Technologies, Inc.), mouse monoclonal anti-CD31 antibody (JC70A, cat. no. GA61061-2, ready-to-use; Dako; Agilent Technologies, Inc.), mouse monoclonal anti-Ki-67 antibody (MIB-1, cat. no. GA62661-2, ready-to-use; Dako; Agilent Technologies, Inc.) and mouse monoclonal anti-smooth muscle actin antibody (1A4, cat. no. GA61161-2, ready-to-use; Dako; Agilent Technologies, Inc.). The final development of the sections was carried out with DAB containing 0.03% H_2_O_2_. Histological and immunohistochemical evaluations were performed using an Olympus BX50 light microscope (Olympus Corporation).

### Treatment with pazopanib

The typical pazopanib regimen at the present institution was as follows. Pazopanib was administered orally once daily upon awakening, initiated at 200 or 400 mg. When adverse events, primarily diarrhea and loss of appetite (anorexia), were deemed tolerable by the treating physician, the dose was increased to 600 mg, with further escalation to 800 mg and continuation at that dose whenever feasible. If 800 mg was not tolerated, treatment was maintained at 600 mg.

## Results

### Patient characteristics

**Figure 1.**
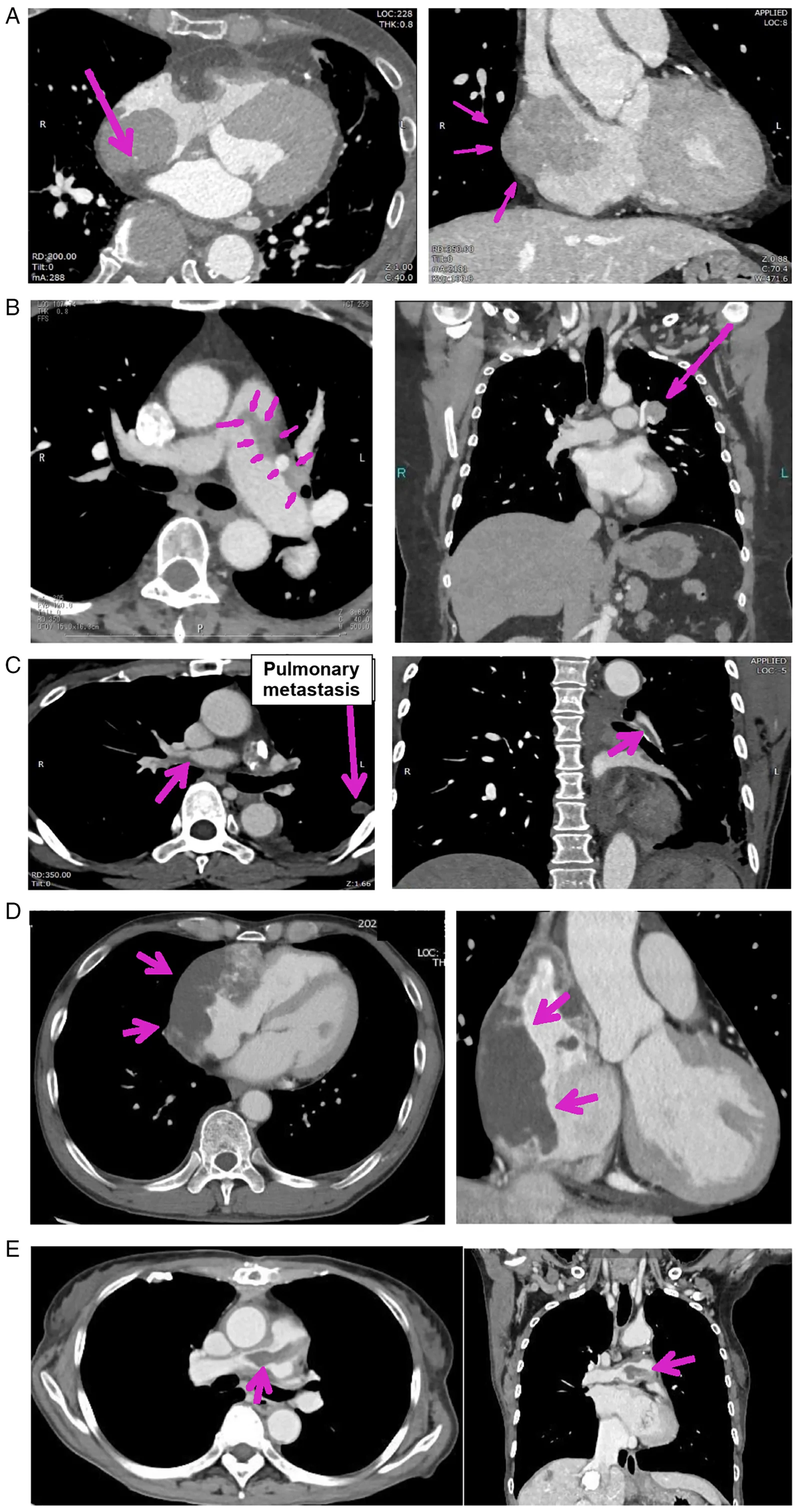
Contrast-enhanced computed tomography imaging. Arrows indicate the primary cardiovascular sarcoma lesion. (A) Case 1: Atrial angiosarcoma (75-year-old man). (B) Case 2: Pulmonary artery sarcoma (intimal sarcoma) (68-year-old woman). (C) Case 3: Pulmonary artery sarcoma (intimal sarcoma) (56-year-old woman). (D) Case 4: Atrial leiomyosarcoma (70-year-old man). (E) Case 5: Leiomyosarcoma (81-year-old woman).

**Figure 2.**
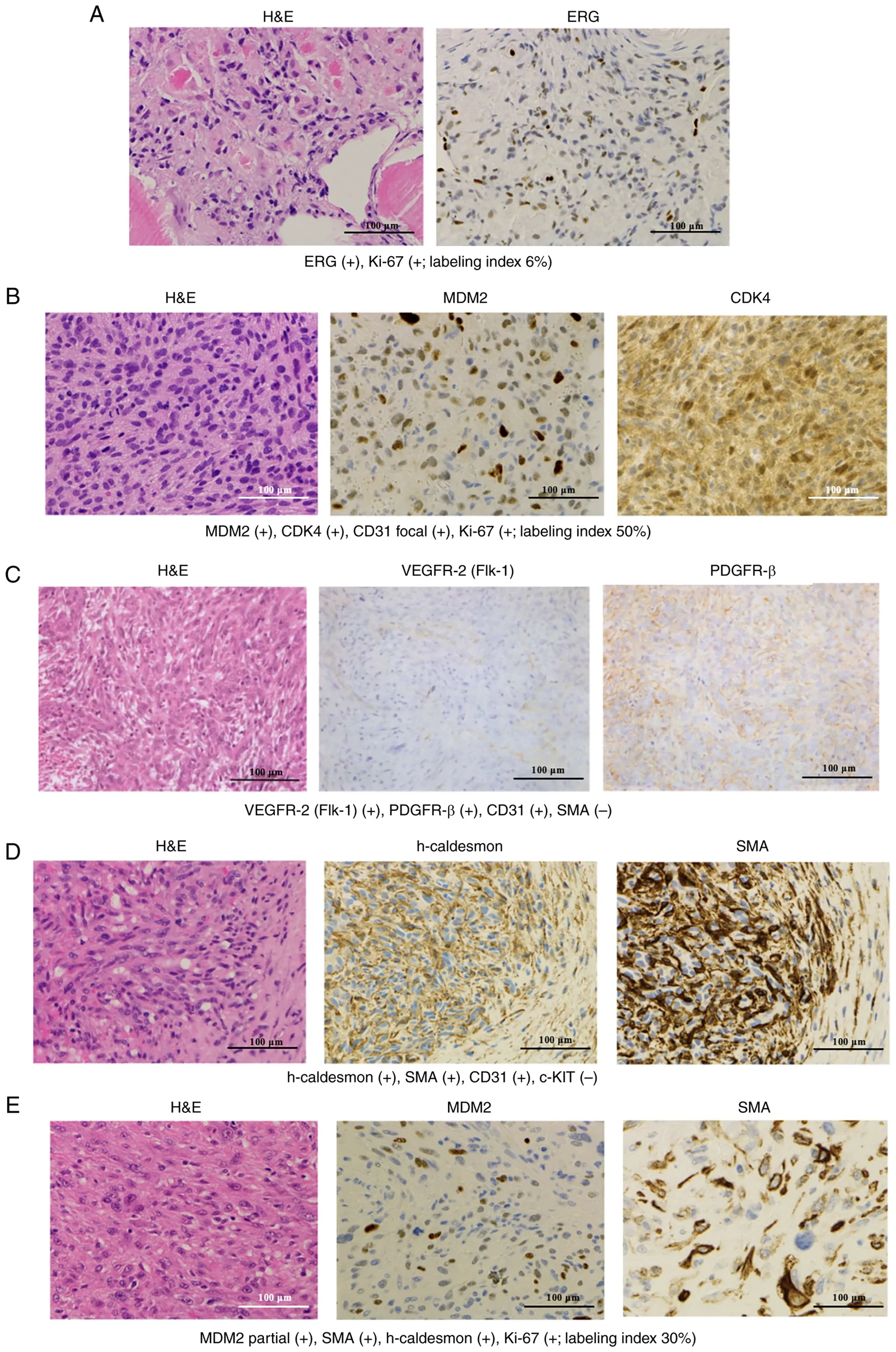
Histopathological and immunohistochemical findings of the tumor lesions. Scale bar, 100 µm. (A) Case 1: Atrial angiosarcoma (75-year-old man). (B) Case 2: Pulmonary artery sarcoma (intimal sarcoma) (68-year-old woman). (C) Case 3: Pulmonary artery sarcoma (intimal sarcoma) (56-year-old woman). (D) Case 4: Atrial leiomyosarcoma (70-year-old man). (E) Case 5: Leiomyosarcoma (81-year-old woman). Representative H&E and immunohistochemical images are shown for each case; the immunohistochemical markers illustrated are (A) ERG, (B) MDM2 and CDK4, (C) VEGFR2 (Flk-1) and PDGFR-β, (D) h-caldesmon and SMA, and (E) MDM2 and SMA. The Ki-67 index was 6% in case 1, 50% in case 2 and 30% in case 5, and is not available for cases 3 and 4. CDK4, cyclin-dependent kinase 4; H&E, hematoxylin and eosin; MDM2, mouse double minute 2; PDGFR-β, platelet-derived growth factor receptor-β; SMA, smooth muscle actin; VEGFR2, vascular endothelial growth factor receptor 2.

**Figure 3.**
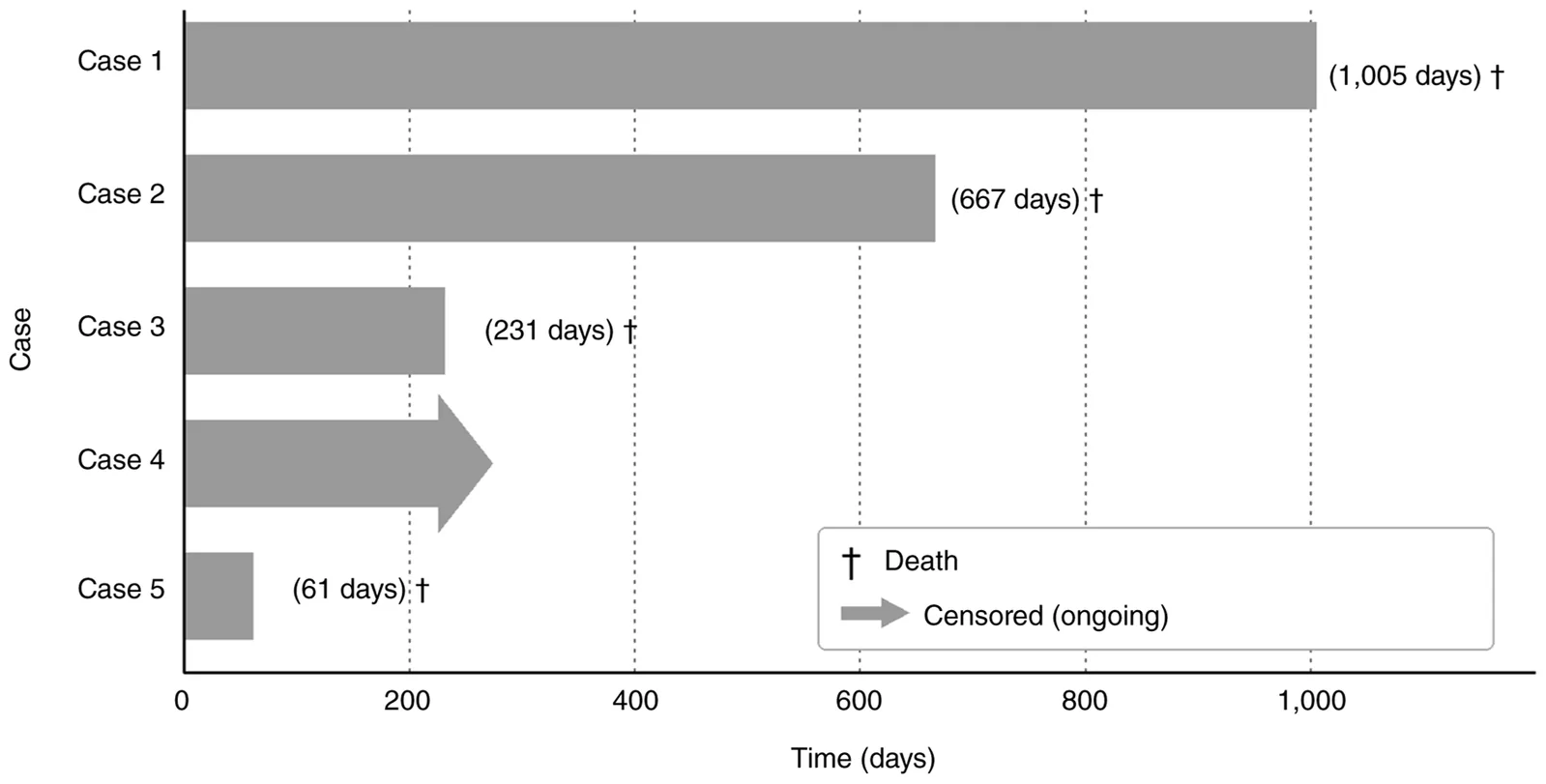
Swimmer plot of pazopanib treatment duration (n=5). Each bar represents the treatment duration for an individual patient. Symbols indicate dose changes, adverse events, and treatment outcomes. The horizontal axis represents follow-up duration in days, and the vertical axis indicates individual cases.

**Figure 4.**
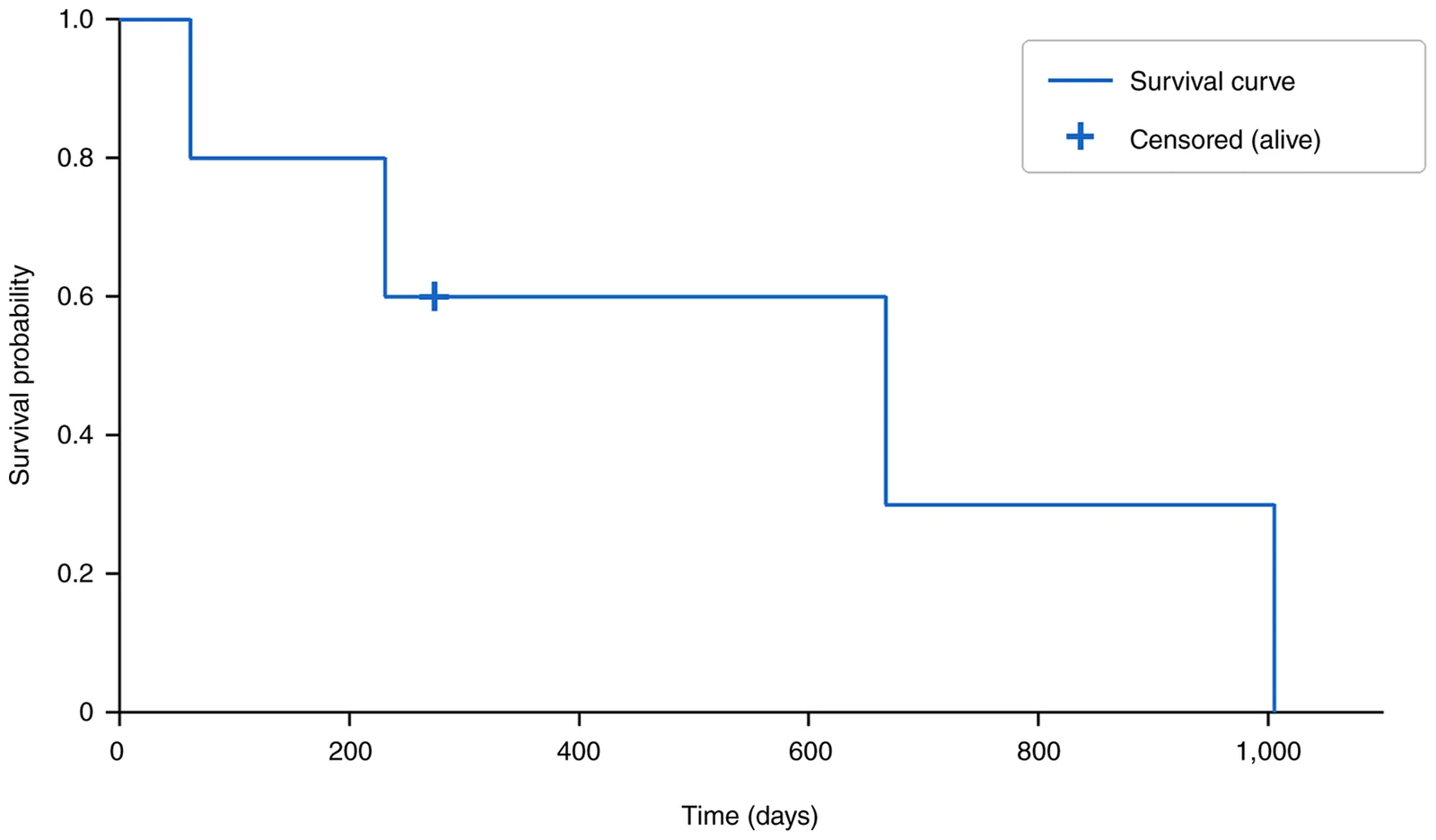
Kaplan-Meier survival curve depicts overall survival over time (n=5). The horizontal axis represents days since pazopanib initiation and the vertical axis represents the estimated survival probability. Stepwise declines indicate death events, whereas the plus symbol denotes a censored patient who was alive at the last follow-up.

Five consecutive patients with unresectable PCS were treated with pazopanib during the study period. Patient characteristics, dose history, and treatment outcomes are summarized in Table I. Contrast-enhanced computed tomography (CT) imaging and histopathological findings for each case are shown in Figs. 1 and 2, respectively. Additional immunohistochemical images for each case are also provided (Fig. S1). The duration of pazopanib treatment and the clinical course for each patient are illustrated in Fig. 3, and overall survival across the cohort is shown in Fig. 4. The cohort comprised three women and two men with a median age of 70 years (range, 56–81 years). Histological subtypes were angiosarcoma in one patient (case 1), intimal sarcoma in two patients (cases 2 and 3), and leiomyosarcoma in two patients (cases 4 and 5). All five patients had an Eastern Cooperative Oncology Group (ECOG) performance status (PS) of 2 or higher at presentation.

### Case 1

A 75-year-old man was referred to the oncology department after multiple pulmonary and atrial masses were detected following multiple cerebral infarctions. The patient had no symptoms other than a mild speech disorder resulting from the cerebral infarctions. Right heart catheterization was performed to evaluate the atrial mass lesion, and a diagnosis of atrial angiosarcoma was made on the basis of catheter aspiration cytology. A needle biopsy was also performed on the lung lesions, and similar pathological findings confirmed multiple pulmonary metastases of angiosarcoma. The patient began treatment with pazopanib at 400 mg, which was gradually increased due to the absence of adverse effects, with a maintenance dose of 800 mg. Although he later complained of mild fatigue and loss of appetite, he continued taking 800 mg of pazopanib. His tumors, including the metastatic lesions, remained in a state of stable disease (SD). Two years and 9 months after the start of treatment, he died suddenly of a cerebral infarction.

### Case 2

A 68-year-old woman was diagnosed with intimal sarcoma at the left pulmonary artery bifurcation and underwent surgical resection. Six months later, she was found to have recurrence and was referred to our oncology department. Her chief complaint was exertional dyspnea, and contrast-enhanced CT revealed a poorly enhancing mass within the right and left pulmonary artery bifurcation. Based on her medical history and imaging findings, she was diagnosed with recurrent intimal sarcoma. As the patient did not wish to undergo reoperation, oral pazopanib was initiated at 200 mg and subsequently increased to 600 mg. Although she experienced adverse effects including loss of appetite, fatigue, and discoloration of body hair, she continued taking 400 or 600 mg for approximately 1 year and 10 months, during which her disease remained stable. However, pazopanib was eventually discontinued because the fatigue became intolerable, and she later died of worsening heart failure.

### Case 3

A 56-year-old woman was referred to the oncology department after being diagnosed with a mass in the left pulmonary artery. Four years earlier, she had undergone surgical resection of a left pulmonary artery mass and was diagnosed with primary left pulmonary artery sarcoma (intimal sarcoma). Following complete resection of the tumor, she received six courses of adjuvant chemotherapy with adriamycin and ifosfamide. The current lesion was diagnosed as an unresectable recurrence of the same disease. Cancer genome profiling testing revealed CDK4 gene amplification. The patient was enrolled in a clinical trial with a CDK4/6 inhibitor, but her disease was deemed refractory within 2 months, and oral pazopanib was initiated at 200 mg. The dose of pazopanib was gradually increased; however, because her fatigue and loss of appetite worsened at doses above 600 mg, maintenance doses of 400 or 600 mg were used depending on her symptoms. Eventually, severe fatigue and suicidal ideation made it difficult for her to continue treatment, and pazopanib was discontinued after 7 months. She subsequently died of heart failure.

### Case 4

A 70-year-old man presented to the cardiology department with a chief complaint of syncope. His electrocardiogram during the episode showed sick sinus syndrome (SSS), and contrast-enhanced CT revealed a mass in his right atrial cavity. An urgent permanent pacemaker was implanted, and a diagnosis of right atrial leiomyosarcoma was made on the basis of aspiration cytology obtained during right atrial catheterization. A metastatic lesion was also identified in the S9 segment of the left lung, adjacent to the rib. As the tumor was unresectable, oral pazopanib was initiated at 400 mg. The adverse effects of fatigue and diarrhea were tolerable, so the dose was increased to 800 mg. Six months after the initiation of pazopanib, the patient developed a pericardial effusion due to malignant (neoplastic) pericarditis and underwent a pericardial window procedure. Pazopanib was temporarily interrupted before and after the operation and resumed one week postoperatively. At present, 8 months after the initiation of pazopanib, the patient continues oral therapy at 800 mg.

### Case 5

An 81-year-old woman presented to the oncology department for treatment of a recurrent leiomyosarcoma in the left main pulmonary artery. Eight months earlier, she had undergone surgery to remove a leiomyosarcoma from the right pulmonary artery, and the current lesion was diagnosed as a recurrence. Her chief complaint was exertional dyspnea. After consultation, oral pazopanib was initiated at 200 mg. Because the adverse effects (fatigue, diarrhea, and hypothyroidism) were tolerable, the dose was increased. She was maintained on alternating 400 and 600 mg for 2 months; however, she discontinued treatment because of severe fatigue, and she later died.

## Discussion

PCS are exceptionally rare and aggressive malignancies that originate from the heart and major blood vessels. While benign tumors such as myxomas constitute the majority of primary cardiac lesions, approximately 25% are malignant, with primary cardiac sarcomas accounting for roughly 88.5% of these malignant cases (2). Among PCS, angiosarcoma is the most prevalent histological subtype, often arising from the right atrium, while intimal sarcoma is also frequently observed, particularly within the pulmonary arteries. Although PCS is often reported in relatively younger patients (median age, 45–50 years), the present cases involved older individuals (56–81 years), suggesting that PCS should also be considered in elderly patients when compatible clinical features are present (3).

The prognosis for PCS remains dismal due to its rapid growth, high rate of local recurrence, and early metastatic spread; therefore the 5-year survival rate is reported to be as low as 11.5% (2). According to a recent systematic review, patients with PCS have median PFS and OS of 5 months [95% confidence interval (CI), 4–7 months] and 12 months (95% CI, 10–16 months), respectively, and patients with unresectable or recurrent disease face even shorter survival times (4).

Clinical manifestations of PCS are often non-specific, frequently leading to delayed diagnosis. Dyspnea is the most common symptom, as observed in cases 2 and 5. However, patients may also present with life-threatening complications such as cardiac tamponade, syncope, or arrhythmias (5). Case 4 exemplifies an acute presentation in which syncope and SSS necessitated urgent permanent pacemaker implantation before the diagnosis of right atrial leiomyosarcoma was established. Furthermore, embolic phenomena are serious complications of cardiovascular sarcomas. While left-sided benign tumors such as myxomas are well known to cause cerebral embolization, patients with primary cardiac sarcomas should likewise be monitored carefully for ischemic stroke. In fact, case 1 initially presented with multiple cerebral infarctions and, despite achieving SD with continued treatment, ultimately died from recurrent cerebral infarction. Although an embolic event related to pazopanib cannot be entirely excluded, the patient had developed cerebral infarction before pazopanib was initiated, and the fatal embolism—part of this same sequence—is more plausibly attributed to the sarcoma itself. Progressive heart failure from pulmonary artery obstruction, as seen in case 2, warrants similar consideration: although pazopanib-related cardiac dysfunction cannot be excluded, the heart failure worsened with tumor progression even after pazopanib was discontinued, again pointing to a tumor-related cause. Other reports have documented cardiac sarcomas causing embolic superior mesenteric artery occlusion, leading to acute mesenteric ischemia (6). Based on these previous reports, the PS at diagnosis is extremely poor in the majority of PCS cases. In fact, all of the present cases had an ECOG PS of 2 or higher and were referred to the present institution because of difficulties in managing their treatment.

For PCS, complete surgical resection remains the therapeutic mainstay, yet it is rarely achievable (estimated in <50% of cases) due to tumor inaccessibility and the involvement of vital cardiac structures (4). When surgery is incomplete or impossible, a multidisciplinary approach involving radiotherapy and systemic therapy becomes essential. Although anthracycline-based regimens have long been the standard for advanced STS, their use in PCS can be constrained by cumulative cardiotoxicity, particularly in patients with intracardiac disease or baseline cardiac dysfunction.

Notably, the EPAZ randomized phase II trial demonstrated that pazopanib was non-inferior to doxorubicin as first-line treatment for metastatic STS in elderly patients (≥60 years), with no significant differences in either PFS [hazard ratio (HR), 1.00; 95% CI, 0.65–1.53] or OS (7). Considering these findings along with the cumulative cardiotoxicity of anthracyclines, it may be worth considering pazopanib as a first-line option from the outset regardless of PS, particularly in patients with cardiovascular sarcoma.

Pazopanib, a multitargeted tyrosine kinase inhibitor, has emerged as an important option for patients with progressive metastatic STS after standard chemotherapy. In the PALETTE phase III trial, pazopanib significantly prolonged median PFS compared with placebo (8). In the present series, two patients achieved durable disease stabilization (>500 days) on pazopanib, supporting its potential role as an outpatient treatment option for selected cases of unresectable or recurrent PCS.

Beyond antitumor activity, pazopanib may offer practical advantages in this setting because it is an oral agent. Unlike intravenous regimens that often require frequent hospital visits for each administration, oral therapy can reduce the number of clinic visits and is frequently manageable even at local community hospitals where the delivery of infusional chemotherapy is difficult. This may lessen patient burden and help to maintain continuity of care. Notably, our previous work in patients with cancer (restricted to sarcoma) suggested that disruption of outpatient follow-up itself is associated with shortened survival (9). From this perspective, selecting an oral regimen that facilitates sustained outpatient management may have clinical value beyond drug efficacy alone.

A major barrier to continued pazopanib therapy is treatment-related toxicity, most commonly diarrhea and fatigue (8). These adverse events often become more prominent with dose escalation, and in the present cohort, cases 3 and 5 required discontinuation due to worsening toxicities during up-titration. While the survival impact of continued therapy cannot be determined from this small series, accumulating evidence indicates that longer duration of pazopanib exposure is associated with improved survival outcomes (10). In addition, among patients who achieved only short-term benefit, approximately 40% reportedly discontinued pazopanib due to toxicity—around four times the rate observed in patients who achieved long-term responses (10). Collectively, these findings underscore that how to keep patients on therapy is a key clinical question when pazopanib is selected.

Therefore, to maximize the likelihood of sustained treatment, careful monitoring and individualized management are essential. Practical measures include tailoring the timing and pace of dose escalation to each patient's tolerance, implementing proactive supportive care for adverse events, and using temporary interruptions when needed rather than forcing uninterrupted dose intensification. In this context, case 4 has remained on pazopanib for 8 months and was successfully escalated to and maintained at 800 mg, even though a pericardial window for malignant pericardial effusion was required 6 months into treatment, with only a brief peri-operative interruption. If tolerability is maintained, continued therapy may provide further clinical benefit, although close monitoring remains warranted.

In conclusion, PCS pose major therapeutic challenges due to their rarity and aggressive clinical course. The present findings suggest that pazopanib can provide meaningful disease control in some patients with unresectable or recurrent disease, allowing for long-term stabilization in an outpatient setting. However, frequent drug-related toxicities necessitate careful monitoring and individualized dose management to maintain therapy as long as feasible. The present study has several limitations, including the small sample size (n=5), the retrospective single-center design, and the absence of formal response evaluation by RECIST. Larger, multi-institutional prospective studies are needed to confirm the role of pazopanib in this rare disease setting.

## Supplementary Material

Supporting Data

## Figures and Tables

**Table I.** Clinicopathological characteristics and treatment outcomes of five patients with primary cardiovascular sarcoma treated with pazopanib.

Case no.	Age, years/sex	Primary site	Histology	Metastasis	Prior treatment	Initial dose, mg/day	Max dose, mg/day	Maintenance dose, mg/day	Duration, months
1	75/M	Right atrium	Angiosarcoma	Lung (multiple)	None	400	800	800	33
2	68/F	Left pulmonary artery	Intimal sarcoma	None	Surgery	200	600	400-600	22
3	56/F	Left pulmonary artery	Intimal sarcoma	None	Surgery; Adriamycin + ifosfamide; CDK4/6 inhibitor trial	200	600	400-600	7
4	70/M	Right atrium	Leiomyosarcoma	Lung (S9)	None	400	800	800	8 (ongoing)
5	81/F	Right pulmonary artery	Leiomyosarcoma	None	Surgery	200	600	400 or 600	2

M, male; F, female. Treatment duration was calculated from the initiation of pazopanib to the date of discontinuation or last follow-up. Dose adjustments were performed according to the tolerability of adverse events as assessed by the treating physician.

## Data Availability

The data generated in the present study may be requested from the corresponding author.

## Abbreviations

CT: computed tomography
ECOG: Eastern Cooperative Oncology Group
OS: overall survival
PCS: primary cardiovascular sarcoma
PFS: progression-free survival
PS: performance status
SD: stable disease
STS: soft tissue sarcoma
