# Supplementary material for: Pazopanib treatment for primary cardiovascular sarcoma: A retrospective observational study and literature review
Source: Oncol Lett. 2026 Aug 21;32(4):474. doi: 10.3892/ol.2026.15829 (PMC13572868; doi:10.3892/ol.2026.15829)

Figure S1. Additional histopathological and immunohistochemical findings of the tumor lesions. Representative immunohistochemical images that are not presented in Fig. 2 are shown for each case. Scale bar, 100 μm. (A) Case 1: Atrial angiosarcoma (75-year-old man); Ki-67 (index, 6%). (B) Case 2: Pulmonary artery sarcoma (intimal sarcoma) (68-year-old woman); CD31 and Ki-67 (index, 50%). (C) Case 3: Pulmonary artery sarcoma (intimal sarcoma) (56-year-old woman); CD31 and SMA. (D) Case 4: Atrial leiomyosarcoma (70-year-old man); CD31 and c-Kit. (E) Case 5: Leiomyosarcoma (81-year-old woman); h-caldesmon and Ki-67 (index, 30%). H&E, hematoxylin and eosin; SMA, smooth muscle actin.

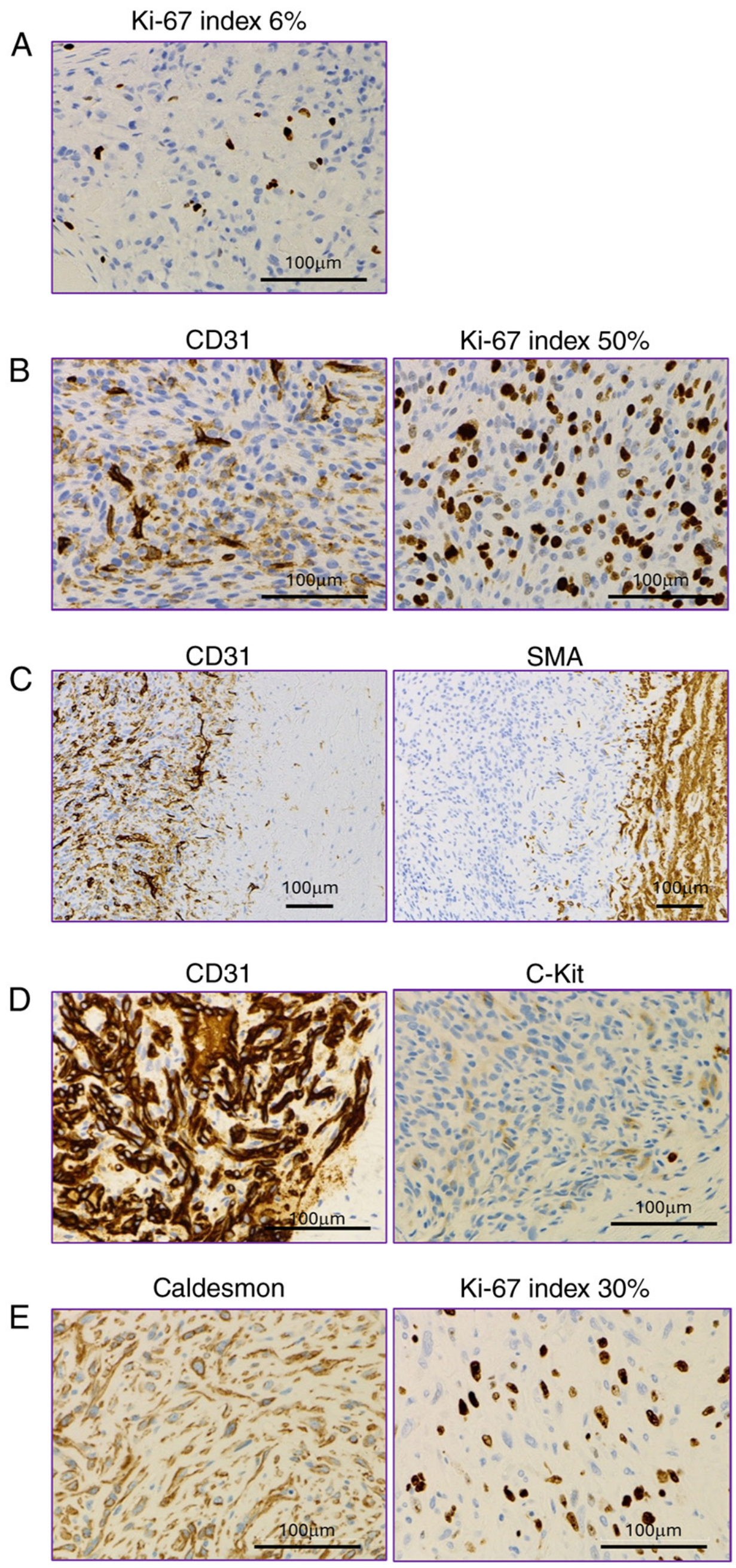

Supplement: Supplementary file 1 [file Supplementary_Data.pdf]
